# Growth mindset and academic outcomes: a comparison of US and Chinese students

**DOI:** 10.1038/s41539-021-00100-z

**Published:** 2021-07-19

**Authors:** Xin Sun, Shaylene Nancekivell, Susan A. Gelman, Priti Shah

**Affiliations:** 1 Department of Psychology, University of Michigan, Ann Arbor, MI USA; 2 Department of Psychology, University of North Carolina Greensboro, Greensboro, NC USA

**Keywords:** Human behaviour, Education, Science in culture

## Abstract

Chinese students are more likely than US students to hold a malleable view of success in school, yet are more likely to hold fixed mindsets about intelligence. We demonstrate that this apparently contradictory pattern of cross-cultural differences holds true across multiple samples and is related to how students conceptualize intelligence and its relationship with academic achievement. Study 1 (*N* > 15,000) confirmed that US students endorsed more growth mindsets than Chinese students. Importantly, US students’ mathematics grades were positively related to growth mindsets with a medium-to-large effect, but for Chinese students, this association was slightly negative. Study 2 conceptually replicated Study 1 findings with US and Chinese college samples, and further discovered that cross-cultural differences in intelligence mindset beliefs corresponded to how students defined intelligence. Together, these studies demonstrated systematic cross-cultural differences in intelligence mindset and suggest that intelligence mindsets are not necessarily associated with academic motivation or success in the same way across cultures.

## Introduction

Decades ago, Stevenson and colleagues^1–6^ argued that Chinese and Japanese students are more likely to attribute academic success to effort over innate ability than US students. These differences in thinking are thought to be early emerging. For example, when listening to learning-related narratives, 4-year-old Chinese children were found to talk more about diligence, whereas their US peers talked more about ability^7^. It has been suggested that such differences may stem from differences in parental practices, as Chinese parents also attribute children’s success to effort more than US parents^5,8,9^. In theory, this emphasis on effort is associated with endorsing a growth mindset^10^. Evidence in support of this link is mainly from Western, Educated, Industrialized, Rich, and Democratic (WEIRD) contexts. Specifically, in WEIRD contexts, students who believe that intelligence is malleable are more likely to attribute academic success to effort, and in turn, succeed academically^10^.

However, there is evidence that conceptualizations of intelligence and their link to achievement may not be universally shared^11^. For example, studies that have directly probed students’ implicit theories of intelligence find that Chinese and Japanese students hold more fixed mindsets compared to their Western counterparts^12,13^. Even ethnic Chinese students growing up in the US develop more growth mindsets compared to Chinese students growing up in China^14^. The fact that Chinese students hold fixed mindsets, yet nonetheless value effort and succeed academically, suggests that there might be systematic differences in how Chinese and US students reason about intelligence. Notably, these systematic differences are often neglected or explained away in favor of supporting a “WEIRD model” of intelligence and achievement. For example, in one such investigation, researchers suggested that their “unexpected” findings were due to the fact that the participants in their study were likely too young to show their predicted pattern, and they suggested that older Chinese students (not sampled) likely would have the predicted more growth mindsets (“there may be a developmental trend with implicit theory orientations (in Chinese children) starting relatively low… and increasing with age…”^14^ p. 122).

In this study, we revisit this potential cultural difference in intelligence mindsets. Specifically, we test whether Chinese students do, in fact, hold more fixed mindsets and examine how these differences are related to potential cross-cultural differences in how intelligence, effort, and achievement are conceptualized. We propose that cultural differences in people’s beliefs about the malleability of intelligence are systematic, and that such differences reflect how Chinese and US lay individuals conceptualize intelligence and its relation with academic achievements. Our first goal is to understand how variations in Chinese and US students’ definitions of intelligence might account for the cross-cultural differences in intelligence mindsets. Prior studies have found that Chinese laypeople emphasize aspects such as reasoning ability, creativity, and memory^15,16^, whereas US individuals highlight practical problem-solving, verbal ability, and social competence^17^. Moreover, recent research has found that individuals’ mindset beliefs are influenced by how intelligence is defined to them^18^. Thus, cultural differences in how intelligence is construed may lead to cultural differences in mindset beliefs.

A second goal of the present study is to better understand the extent to which mindsets are associated with effort attributions and academic performance. We propose that although growth mindsets are related to these academic outcomes in US students, this may not apply to Chinese students to the same extent. In other words, to Chinese students, there may be less of a tight link between intelligence mindsets (on the one hand) and motivations to engage in effortful study and academic performance (on the other hand). Cross-cultural research has found that when asked to write down terms related to “learning,” US individuals often mention the importance of intelligence in learning outcomes, whereas Chinese individuals rarely discuss intelligence^2^. Prior studies have found that Chinese students believe that school performance is significantly more malleable than intelligence^19^. This suggests that, for Chinese students, whether intelligence can be changed may not have bearing on whether they work hard at school. Indeed, working hard is considered a virtue in and of itself, regardless of a person’s intellectual abilities^20^. Thus, the pattern of fixed intelligence mindsets and positive educational outcomes turns out not to be contradictory after all. By testing the present proposals, we build on prior work by being the first to directly test the nuanced ways in which culture might influence how intelligence is conceptualized (i.e., growth or fixed; fluid or crystallized)^18^ and how potential differences in these conceptualizations, which have often been overlooked in prior work^12–14^, might influence educational outcomes (i.e., test scores; attributions on academic achievement).

To test these proposals, we first explored whether and how Chinese and US students differed in their beliefs about intelligence and their associations with academic outcomes, by using the 2018 Programme for International Student Assessment (PISA)^21^. Specifically, Study 1 tested whether Chinese (*N* = 11,979) and the US (*N* = 4,663) middle-school students endorsed different malleability views of intelligence and whether these views were associated with mathematics performance in each sample. To our knowledge, this is the first cross-cultural comparison of mindset beliefs using such a large-scale dataset. This investigation is thus well-suited to test how the mindset theory characterizes students in different cultural contexts and how mindset as a motivational factor is associated with academic outcomes in different educational systems.

In Study 2, we aimed to conceptually replicate Study 1 results by asking college students (US: *N* = 190; China: *N* = 171) about their mindset beliefs of intelligence and effort vs. ability attributions in school- and expert-level mathematics achievements. We assessed the cultural differences in students’ mindset endorsements, as well as how mindsets are related to academic attributions differently. Study 2 further examined how participants’ spontaneous definitions of intelligence were associated with beliefs about their malleability in these two cultures. We took two approaches. First, students were asked to spontaneously define intelligence in their own words. These definitions were then coded based on whether they reflected fluid and/or crystallized intelligence. Second, we manipulated definitions of intelligence by providing specific definitions to the participants (i.e., a fluid definition and a crystallized definition) and we probed students’ mindsets of each definition.

## Results and discussion

### Study 1

#### Do Chinese students endorse a more fixed mindset than US students?

Table 1 shows the frequencies and percentages of the four mindset responses for each sample. Notably, 68.39% of the US students regarded intelligence as malleable (choosing “1-Strongly Disagree” or “2-Disagree”), compared to only 55.61% of the Chinese students. Regression analysis yielded significance for country, *β*_country_ = 0.24, SE_country_ = 0.02, *t* = 11.59, 95% confidence interval (95% CI) = (0.20, 0.28). These results showed that Chinese students held significantly more fixed mindsets than US students.

**Table 1:** Chinese and US students’ response frequency and percentage estimate to the mindset item.

Response to the mindset item	China (B-J-S-G) (*N* = 11,979)			US (*N* = 4663)		
	Frequency	Percentage (%)	SE	Frequency	Percentage (%)	SE
1-Strongly disagree (growth)	2141	18.86%	0.63	1414	30.17%	0.71
2-Disagree (somewhat growth)	4199	36.75%	0.65	1740	38.22%	0.88
3-Agree (somewhat fixed)	4351	35.35%	0.62	1046	22.41%	0.58
4-Strongly agree (fixed)	1288	9.04%	0.36	463	9.20%	0.49

The mindset statement is “Your intelligence is something about you that you can’t change very much”.

#### Are mindsets associated with mathematics performance in each student sample?

Regression analysis was conducted using country and mindset responses to predict mathematics performance. As predicted, country and mindset responses significantly predicted mathematics scores, *β*_country_ = 37.89, SE_country_ = 6.30, *t* = 6.01, 95% CI = (25.54, 50.25); *β*_mindset_ = −59.40, SE_mindset_ = 3.77, *t* = −15.77, 95% CI = (−52.02, −66.78). Importantly, their interaction also yielded significance, *β*_country*mindset_ = 34.16, SE_country*mindset_ = 2.28, *t* = 14.99, 95% CI = (29.69, 38.62), suggesting that mindset is associated with mathematics achievements differently across the two countries.

To draw out the effects of mindset on mathematics achievement, six pairwise comparisons of mathematics scores by mindset responses were conducted for each country. Table 2 presents score differences and the corresponding effect sizes. For Chinese students, those who held fixed mindsets had higher mathematics scores than those with relatively more growth mindsets (*M*_score difference_ = 10.51–24.86), although this association yielded small effect sizes (*d* = 0.13–0.31). For US students, those who held fixed mindsets had lower mathematics scores than those with relatively more growth mindsets (*M*_score difference_ = −22.64 to −72.78) and this association yielded moderate to large effect sizes (*d* = −0.25 to −0.86).

**Table 2:** Pairwise comparison of PISA math score by mindset responses of Chinese and US students.

Pairwise comparison group	China (B-J-S-G) (*N* = 11,979)		US (*N* = 4663)	
	Mean score difference (*M*_diff_)	Effect size (*d*)	Mean score difference (*M*_diff_)	Effect size (*d*)
4 > 3 “Fixed” > “Somewhat fixed”	10.51	0.13	−22.64	−0.25
4 > 2 “Fixed” > “Somewhat growth”	24.50	0.31	−72.78	−0.86
4 > 1 “Fixed” > “Growth”	24.86	0.31	−71.45	−0.84
3 > 2 “Somewhat fixed” > “Somewhat growth”	13.99	0.17	−50.14	−0.55
3 > 1 “Somewhat fixed” > “Growth”	14.35	0.18	−48.81	−0.53
2 > 1 “Somewhat growth” > “Growth”	0.36	0.00	1.33	0.02

**Fig. 1:**
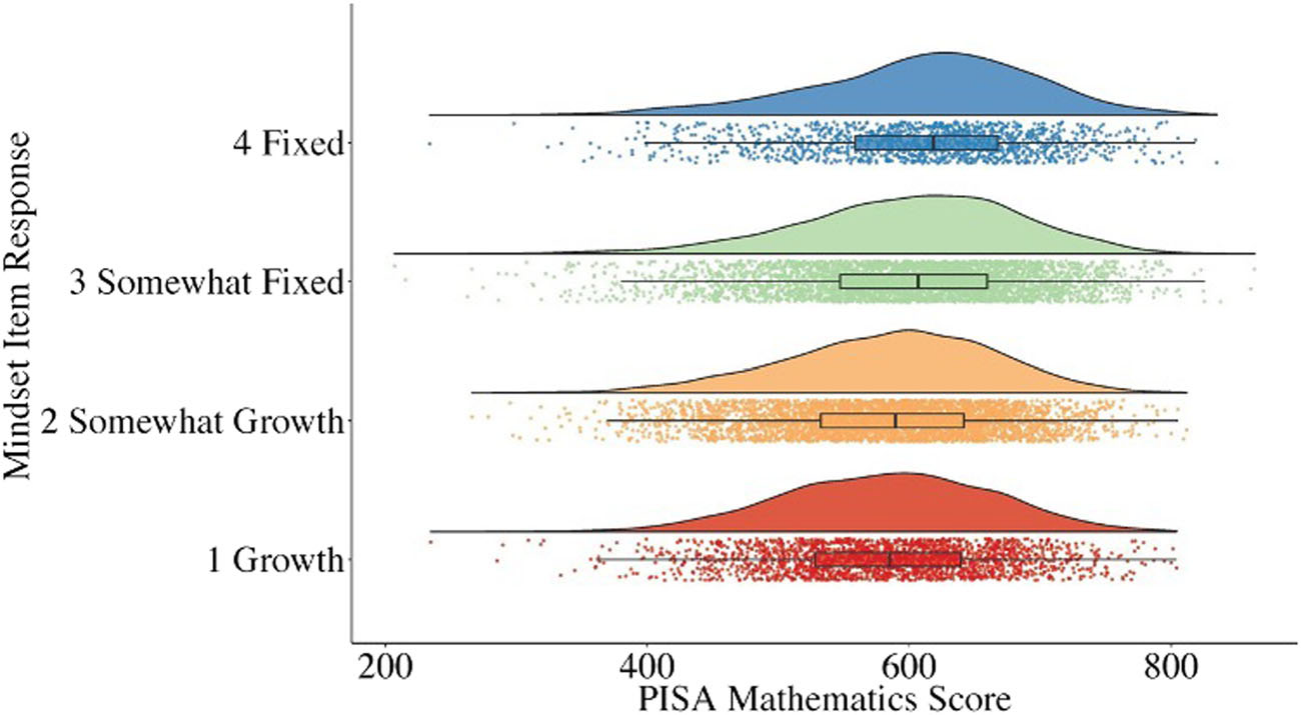
Distribution of mathematics score by mindset response in the Chinese (B-J-S-G) sample. The bolded line inside each boxplot indicates the median mathematics score by mindset responses. Each box covers the middle 50% of the whole score distribution (i.e., the interquartile range, IQR). The line of the boxplot covers the middle 99.3% of the whole score distribution (i.e., 1.5 IQR beyond each end of the box).

**Fig. 2:**
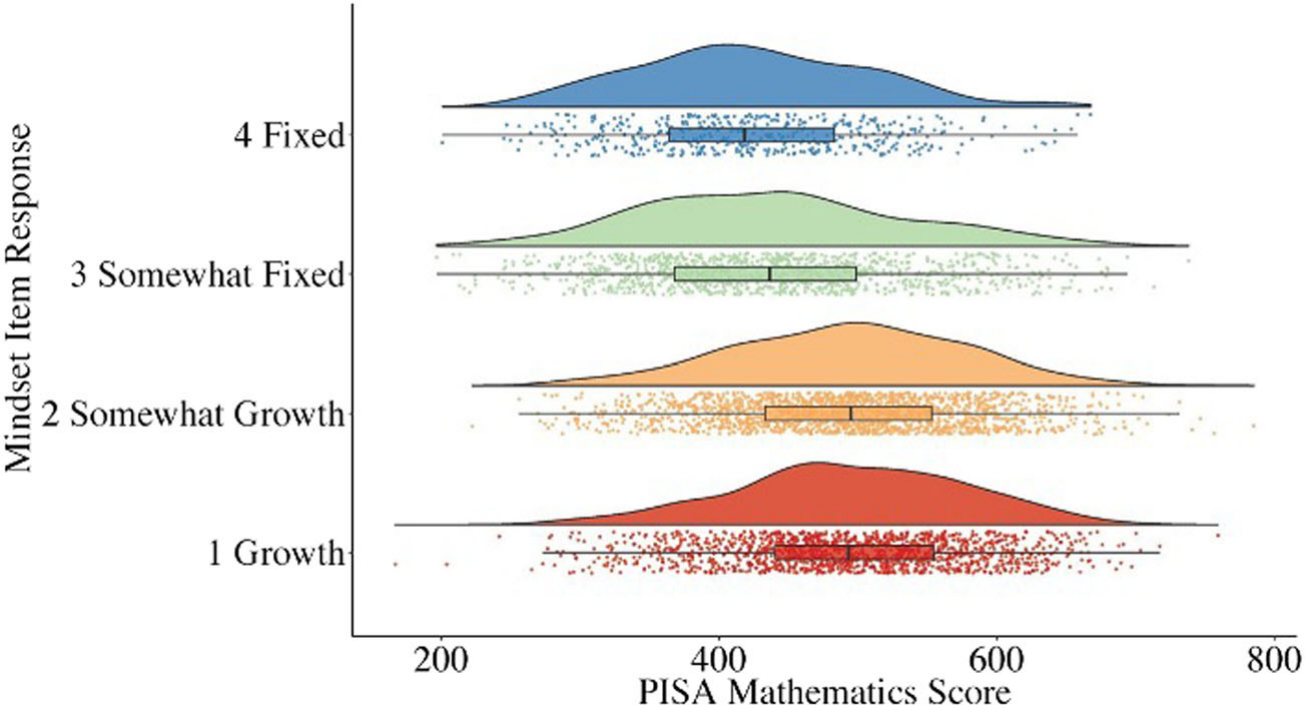
Distribution of mathematics score by mindset response in the US sample. The bolded line inside each boxplot indicates the median mathematics score by mindset responses. Each box covers the middle 50% of the whole score distribution (i.e., the interquartile range, IQR). The line of the boxplot covers the middle 99.3% of the whole score distribution (i.e., 1.5 IQR beyond each end of the box).

We next mapped out distributions of PISA mathematics scores by mindset responses for each country (Figs. 1 and 2). Ten graphs were drawn for the ten plausible values for each group. As all ten graphs showed the same pattern, for simplicity, Figs. 1 and 2 present the graphs with plausible value 1 only (all graphs can be found in Supplementary Figs. 1 and 2). Three elements were presented to depict mathematics score distributions for each mindset item response as follows: a violin plot showing the probability density at each mathematics score point; a jitter plot showing individual point distributions; and a boxplot capturing the median (the bolded line inside each box) and the interquartile range (the two ends of the box indicate quartile 1 and 3).

Altogether, as expected, US students’ mathematics performance was tightly associated with how they reasoned about intelligence, such that students performed better in mathematics if they endorsed a more malleable view of intelligence. For Chinese students, in contrast, fixed mindsets were slightly associated with higher mathematics scores. It should be noted that the effect for US students was medium-to-large, but the effect for Chinese students was small.

### Study 2

Table 3 shows descriptive statistics of the implicit theories of intelligence (i.e., the self-defined intelligence mindsets), effort-ability Attribution measures, and implicit theories of fluid and crystallized definitions of intelligence for each country.

**Table 3:** Descriptive statistics of intelligence mindset and effort-ability attribution.

Survey	Variable	The Chinese sample	The US sample
		*M* (SD)	*M* (SD)
Implicit Theories of Intelligence	Mindset of intelligence	3.08 (0.83)	4.12 (1.09)
	Mindset of fluid intelligence	3.38 (0.90)	3.81 (1.16)
	Mindset of crystallized intelligence	4.39 (0.88)	4.74 (0.94)
Effort-Ability Attribution	School-level achievement	5.53 (1.09)	4.85 (1.14)
	Expert-level achievement	2.55 (1.09)	3.44 (1.38)

The Implicit Theories of Intelligence measure used a 6-point Likert scale, with higher scores indicating a more growth mindset. The Effort-Ability Attribution measure used a 7-point Likert scale, from 1-Pure ability to 7-Pure effort.

#### Do Chinese students endorse a more fixed intelligence mindset but more effort-attribution than US students? Are mindsets associated with attributional outcomes more in the US than in the Chinese sample?

Consistent with Study 1 results, the Study 2 Chinese sample (*M* (SD) = 3.08 (.83)) endorsed a less growth intelligence mindset than the US sample (*M* (SD) = 4.12 (1.09)), *t*(358) = −10.08, *p* < 0.001, *d* = 1.07. At the same time, the Chinese sample (*M* (SD) = 5.53 (1.09)) endorsed a significantly more effort-oriented attribution on school-level achievement compared to the US sample (*M* (SD) = 4.85 (1.14)), *t*(358) = 5.83, *p* < 0.001, *d* = 0.61. However, as for the expert-level achievement, the Chinese sample (*M* (SD) = 2.55 (1.09)) was more ability-oriented than the US sample (*M* (SD) = 3.44 (1.38)), *t*(358) = −6.72, *p* < 0.001, *d* = 0.71. These results replicated findings from Study 1 that Chinese students hold more fixed intelligence mindsets, but still attribute effort towards school academic achievement, and this attribution does not extend to more advanced career-level achievements.

Correlations between intelligence mindset and effort-ability attribution in school-level academic achievement were significant in the US sample (*r* = 0.22, *p* = 0.003), but not in the Chinese sample (*r* = 0.10, *p* = 0.196; *Z* = 1.16, *p* = 0.123). Intelligence mindset correlated with expert-level academic achievement in both groups, with a moderate effect in the US sample (*r* = 0.33, *p* < 0.001) and a small effect in the Chinese sample (*r* = 0.15, *p* = 0.048; *Z* = 1.80, *p* = 0.036). Intelligence mindset correlated with the overall effort-ability attribution of academic achievement in both groups, again with a moderate effect in the US sample (*r* = 0.41, *p* < 0.001) but small effect in the Chinese sample (*r* = 0.18, *p* = 0.016; *Z* = 2.38, *p* = 0.009). These results showed that intelligence mindsets in the Chinese sample were not as strongly related to motivational outcomes in educational contexts compared to that in the US sample, which echoed findings from Study 1.

#### Do Chinese and US students demonstrate differences in mindsets of self-defined intelligence, and the intelligence defined to them (fluid and crystallized definitions)?

**Fig. 3:**
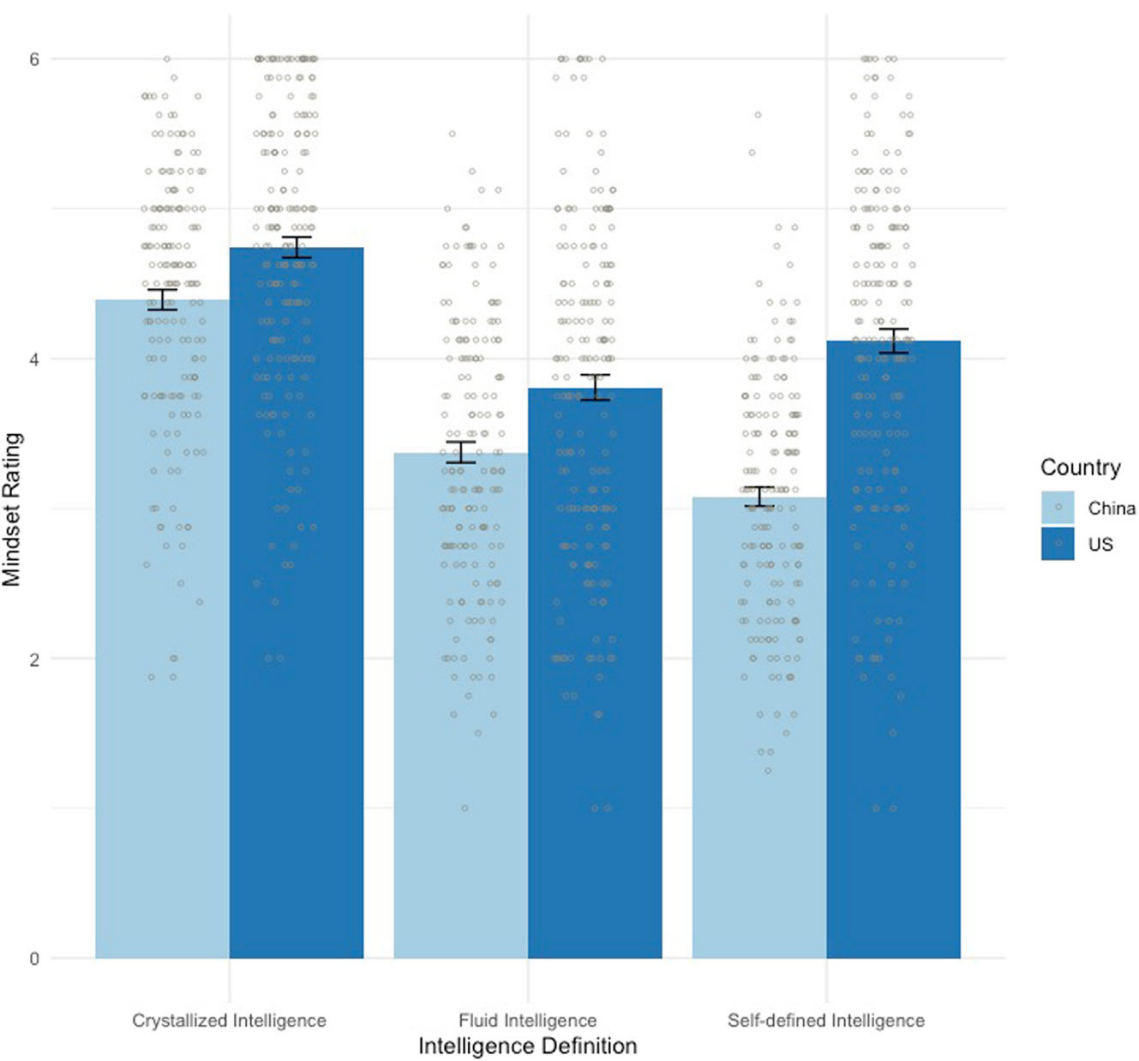
Mindsets of self-defined, fluid, and crystallized intelligence (with SE error bars) by country. Mindset ratings were on a 1–6 Likert scale. Each bar indicates the mean mindset rating with error bars showing the SDs. A larger mean mindset value indicates a more growth mindset.

A 2 (country) × 3 (self-defined intelligence, fluid intelligence, and crystallized intelligence) mixed analysis of variance (ANOVA) was conducted to compare mindset beliefs between the two samples (Fig. 3) and different definitions of intelligence. The two main effects and the interaction were all significant: the country main effect, *F*(1, 358) = 55.88, *p* < 0.001, *η*^*2*^ = 0.14; the intelligence definition main effect, *F*(2, 716) = 205.58, *p* < 0.001, *η*^*2*^ = 0.37; the interaction, *F*(2, 716) = 23.08, *p* < 0.001, *η*^*2*^ = 0.06.

To draw out the intelligence definition main effect, three post hoc pairwise comparisons between the Chinese and US samples were conducted for self-defined, fluid, and crystallized intelligence (*p*-adjusted = 0.017). In all three cases, Chinese students endorsed a more fixed intelligence mindset rating than the US students: self-defined intelligence (presented above); fluid intelligence, *t*(358) = −3.92, *p* < 0.001, *d* = 0.42, and crystallized intelligence, *t*(358) = −3.63, *p* < 0.001, *d* = 0.38.

To draw out the interaction, two sets of post hoc pairwise tests among the three definitions of intelligence were conducted for each country (*p*-adjusted = 0.017). The US sample rated fluid intelligence as more fixed than self-defined intelligence (*t*(188) = −5.10, *p* < 0.001, *d* = 0.28) and crystallized intelligence (*t*(188) = −10.11, *p* < 0.001, *d* = 0.88); and self-defined intelligence was rated as more fixed than crystallized intelligence (*t*(188) = −5.10, *p* < .001, *d* = .61). However, self-defined intelligence was rated as the most fixed in the Chinese sample, as compared to fluid intelligence (*t*(170) = −4.21, *p* < 0.001, *d* = 0.34) and crystallized intelligence (*t*(170) = −16.32, *p* < 0.001, *d* = 1.54). Moreover, fluid intelligence was believed to be more fixed than crystallized intelligence (*t*(170) = −12.16, *p* < 0.001, *d* = 1.14).

#### Do spontaneous intelligence definitions reflect cross-cultural differences in mindset?

Table 4 shows the frequencies and percentages of each sample’s spontaneous intelligence definition by coding category (i.e., number and percentage of definitions that reflect “only fluid intelligence” (fluid-only definition), “both fluid and crystallized intelligence” (combined definition), “only crystallized intelligence” (crystallized-only definition), and “neither of the two”).

**Table 4:** Frequency and percentage of intelligence definitions by coding category.

Category of intelligence definition	The Chinese sample			The US sample		
	Frequency	Percentage	Mindset	Frequency	Percentage	Mindset
Only fluid intelligence	62	36.3%	2.96 (0.85)	51	27.0%	3.73 (1.10)
Both fluid and crystallized intelligence	53	31.0%	3.15 (0.73)	39	20.6%	4.07 (1.18)
Only crystallized intelligence	16	9.4%	3.18 (0.89)	77	40.7%	4.46 (0.88)
Neither fluid or crystallized intelligence	40	23.4%	3.12 (0.89)	22	7.6%	3.91 (1.30)
Full sample	171	100.0%	3.08 (0.83)	189	100.0%	4.12 (1.09)

**Fig. 4:**
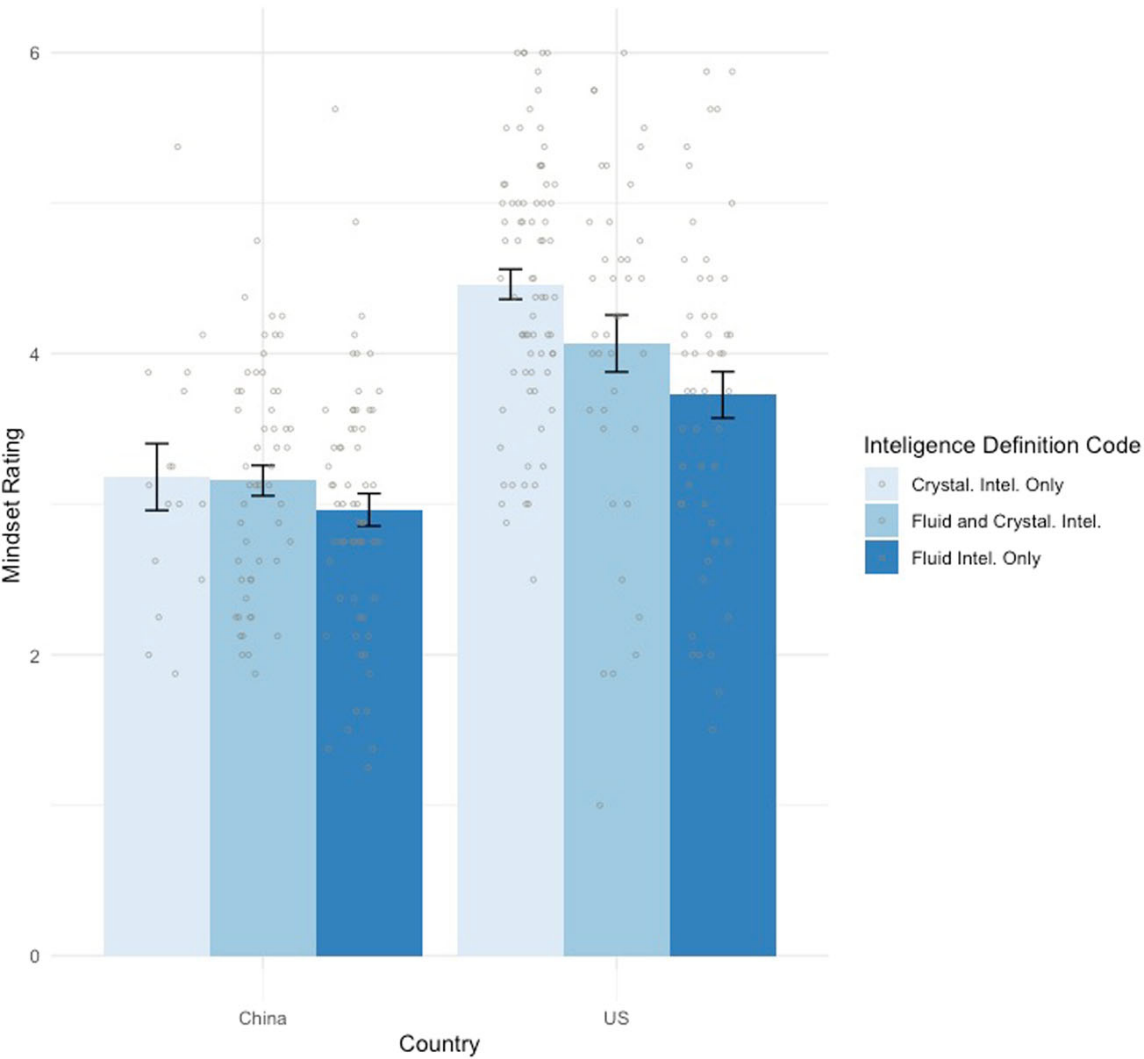
Mindsets of intelligence (with SE error bars) by self-definition code by country. Mindset ratings were on a 1–6 Likert scale. Each bar indicates the mean mindset rating with error bars showing the SDs. A larger mean mindset value indicates a more growth mindset.

A 2 (country) × 3 (intelligence self-definition code) between-subjects ANOVA was conducted to test how spontaneous intelligence definition relates to mindset ratings (Fig. 4). Both main effects were significant, yet the interaction was not: the country main effect, *F*(1, 292) = 64.11, *p* < 0.001, *η*^*2*^ = 0.18; the type of intelligence definition main effect, *F*(2, 292) = 5.11, *p* = 0.007, *η*^*2*^ = 0.03; the interaction, *F*(2, 292) = 1.38, *p* = 0.254, *η*^*2*^ < 0.01.

To map out the main effect of intelligence self-definition code, three post hoc pairwise tests were conducted to compare mindsets between participants with different definitions of intelligence (*p*-adjusted = 0.017). Results showed significant mindset differences between participants with fluid-only (*M* (SD) = 3.31 (1.04)) and crystallized-only (*M* (*SD*) = 4.24 (1.00)) intelligence definition codes, *t*(204) = −6.54, *p* < 0.001, *d* = 0.92; and between participants with combined (*M* (SD) = 3.54 (1.10)) and crystallized-only intelligence definition codes, *t*(183) = −4.64, *p* < 0.001, *d* = 0.68. Mindset ratings did not show a significance between participants with fluid-only and combined intelligence definition codes, *t*(203) = −1.61, *p* = 0.11, *d* = 0.23. These results suggest that, across the two samples, how intelligence was conceptualized in these students made a difference in their mindsets of intelligence. Specifically, a more crystallized self-definition of intelligence is associated with a more growth mindset of intelligence.

A *χ*^2^-test was then conducted to test whether the two samples differed by intelligence category. We removed participants whose intelligence definition codes were in the “neither fluid nor crystallized intelligence” category, because their responses did not incorporate either of the two types of intelligence. The result was significant: *χ*^2^ (2) = 39.44, *p* < 0.001. Therefore, Chinese students incorporated relatively more fluid intelligence and less crystallized intelligence in their spontaneous definitions of intelligence, and this corresponded to their more fixed intelligence mindset.

## Discussion

Contrary to a commonly held view^7,22^, we find across two studies that US students rated intelligence as more malleable than Chinese students. Moreover, mindset beliefs of intelligence might mean different things to students in these two cultures. In Study 1, growth mindsets were associated with higher mathematics grades for US students, with a medium-to-large effect. However, the association was in fact slightly negative for Chinese students. For US students, how they think about the malleability of intelligence is closely tied to their schoolwork performance. In contrast, such beliefs might not matter to Chinese students’ academic outcomes to the same extent. This speaks to the seeming contradiction that Chinese students hold a more fixed mindset about intelligence and at the same time have better school performance. Notably, these results were obtained both in a large international assessment of over 15,000 middle-school students and a more in-depth analysis of the reasoning of two college student samples.

Study 2 found evidence to support the two proposals for the puzzling observations that Chinese students hold more fixed intelligence mindsets than US students, and at the same time, they attribute academic success to effort. First, students in the two cultures conceptualized the construct of intelligence in different ways. Specifically, US students were much more likely to define intelligence either exclusively or nearly exclusively in terms of crystallized (not fluid) skills. Significantly more Chinese students included only fluid (not crystallized) skills in their own intelligence definitions. Research has shown that people’s mindset beliefs are significantly more fixed when intelligence is defined in a fluid as compared to a crystallized way^17^. Therefore, US students’ higher endorsement of growth mindsets seems to reflect their definitions of intelligence as more crystallized, which is seen as more malleable.

The second proposal involves how mindset relates to school academic outcomes. Consistent with Study 1, Study 2 found that growth mindsets were positively associated with attributing mathematics achievements more to effort than innate ability in the US sample, but not as strong in the Chinese sample. These results again suggest that the mindset of intelligence does not matter to Chinese students’ views about academic achievement as much as that to US students. In addition, the Chinese sample was significantly more likely to attribute success in school to effort across the board, compared to the US sample. The differences in beliefs between Chinese and US students are likely rooted in how each culture conceptualizes learning and intelligence. In prior work, when reasoning about learning, US individuals tend to describe intelligence as a cause for learning outcomes; however, Chinese individuals do not emphasize intelligence, but instead reason about how learning and achievement are important to fulfill purposes of life^2,23^. Importantly, to Chinese individuals, everyone “can learn to their fullest potential^23^,” regardless of their intelligence level. As such, we found similar themes in our work. We found that Chinese students devote significant amounts of effort to schoolwork despite their fixed mindset about intellectual abilities and they believe school success is attainable through hard work^24^. Intelligence is relatively fixed and it determines one’s potential; however, one has to invest efforts to learn and achieve their potential^23^.

The current research is consistent with a growing body of research that finds mixed results regarding how mindset beliefs about intelligence are related to academic achievement^25–29^. Notably, recent research has found that the classic mindset intervention paradigm was not effective on a sample of *N* = 624 Chinese students, yielding consistent results with the current investigation^28^. Although fostering a growth mindset appears beneficial for some people and contexts, this is not a simple or uniform effect. The present findings indicate that it is important to be careful in drawing conclusions regarding the implications of growth mindsets^30^. Future research should acknowledge the complexity of these and related beliefs, and seek to further understand the nuances of mindset beliefs in motivation and academic achievement.

Our results particularly highlight the importance of considering cultural context in motivation research. Growth mindsets about intelligence are not universally associated with beliefs about the role of effort in academic success, nor in academic performance. Prior evidence has been rooted primarily in the US and other western societies, and our Chinese data reveal a strikingly different pattern. Specifically, Chinese students endorse fixed mindsets about intelligence but invest much effort and outperform US students, and Chinese students do not link the malleability of intelligence to school academic achievement as tightly as US students. Rather, it is the “mindset” of school performance that matters more to Chinese students’ school outcomes. Researchers have overly focused on this nebulous idea of “intelligence mindset”, which does not play the same causal role in everyone’s belief systems. Future motivation research should consider cultural belief systems—not only about the general mindset construct, but also on what mindsets are regarding, such as intelligence or school performance—when researching in different populations.

Another implication of the present work is that there may actually be two routes to motivation intervention based on mindset theory. One oft-taken route is working to increase beliefs about malleability, yet generally, this effort has small and limited effects on achievement^28^. An alternative approach might be to address the belief that the malleability of intelligence is the key to academic achievement. This aligns with what Ng and Wei^9^ noted: Chinese parents expect their children to invest effort at school “regardless of whether they are gifted”. Rather than emphasizing the malleability of intelligence and its role in academic achievement, interventions can focus on the malleability of mastering school subjects and directly promoting a more effort-oriented attribution to school performance. In this case, students can be motivated to invest effort at school even if they do not hold a growth view of intelligence. A related message might be to emphasize that school success is only modestly related to intelligence per se. After all, students in both cultures view effort as more important than ability for school-level achievement. Reminding students of this belief, in theory, could be more impactful than focusing on intelligence.

Although the present research for the first time investigated cross-cultural variations in intelligence mindset, it still has many limitations, which yield several future directions. First, given the broad scope of participating countries in PISA, future analysis can go beyond the United States and China, and draw out the nuances of intelligence beliefs and academic achievement in other cultures. Second, Study 2 coded intelligence definition within the fluid-crystallized framework, which is not inclusive of how individuals conceptualize intelligence (i.e., 23% of the Chinese students in Study 2 included neither fluid nor crystallized components in their definitions). Studies should further draw out such cultural specifics.

The current investigation is the first that addressed the cultural paradox of Chinese and US students’ mindset beliefs and their associations with academic outcomes. Although Chinese students value effort and succeed academically, they are more likely to endorse a fixed view of intelligence than US students. We demonstrated two explanations for this cultural difference. First, Chinese and US students conceptualize intelligence differently. Second, whereas their growth mindsets are related to motivational beliefs and academic outcomes for US students, they are much less so for Chinese students. Our research suggests that the association between beliefs about intelligence and success is not universal.

## Methods

### Study 1

Study 1 examined cultural differences in mindset beliefs of intelligence using the PISA dataset^21^. PISA is a large-scale test that measures 15-year-old students’ academic performance in mathematics, reading, and science in countries across the world. In addition to academic tests, it includes questionnaires that assess socio-emotional and attitudinal constructs. The most recent wave was administered in 2018 and the questionnaire added an item for the first time on mindset beliefs^21^.

### Participants

Study 1 examined data from the US and Chinese samples of PISA 2018. The US sample included *N* = 4663 participants from 175 schools across the country (49.6% girls), excluding those with missing values (*N* = 175). For the Chinese sample, we included those students from mainland China, specifically from the provinces of Beijing, Jiangsu, Shanghai, and Guangdong (B-J-S-G). Taking out missing values (*N* = 79), the final Chinese sample included *N* = 11,979 participants from 362 schools (47.9% girls).

### Measures

#### Mathematics achievement

The mathematics test involves four broad content categories: change and relationships, space and shape, quantity, and uncertainty and data. Specific content topics under these categories include functions, algebra expressions, equations and inequalities, relationships within and among geometrical objects, data variability, and so on^21^. For each individual student, there are ten estimated plausible values (item labels appeared in the dataset: *PV1MATH* to *PV10MATH*) to represent their mathematics performance^31^.

#### Mindset of intelligence

Students’ mindset beliefs of intelligence were assessed with one questionnaire item (item label appeared in the dataset: ST184^21^). Students were asked to rate the extent to which they agree to the following statement: “Your intelligence is something about you that you can’t change very much”. This item is identical to the second item of the Implicit Theories of Intelligence Questionnaire (ITIQ)^10^. The item was answered on a four-point scale from 1-Strongly disagree to 4-Strongly agree. These responses can be interpreted as 1-Growth, 2-Somewhat growth, 3-Somewhat fixed, and 4-Fixed.

### Data analysis plan

Data analysis was conducted with the R package intsvy. The package produces unbiased estimates by following standard data analysis procedures as suggested by PISA data analysis manual^31^. These procedures account for students’ demographic information, school-level effects, and apply weight replicates to variables of interest^31^.

It would not be meaningful to interpret our results based on *p*-values alone using such a large-sample dataset (almost all *p*s will be significant due to the large degrees of freedom). Instead, best practices^32^ suggest analyzing any data with such large samples (*N* > 10,000) by: reporting effect sizes, reporting CIs, and interpreting visualizations of data distribution and variation. As such, Study 1 focuses on these indicators.

We first performed a regression analysis to examine whether the country (China and United States) is significantly associated with mindset responses. We specifically looked at the 95% CI of the country coefficient, as well as the mindset response distribution as indicated by the percentage of each response by country. Second, to investigate how mindset and country interact to predict mathematics performance, we conducted regression analysis using country, mindset response, and their interaction, to predict mathematics performance.

### Study 2

In Study 1, US students embraced a more growth mindset than Chinese students. In addition, a growth mindset means differently to US and Chinese students: it is positively associated with higher mathematics performance in US students but not in Chinese students. Study 2 aimed to replicate Study 1 findings and further assessed how differences in definitions of intelligence may account for such cultural differences.

We asked participants to spontaneously define intelligence and coded whether their spontaneous definitions reflected fluid or crystallized intelligence. We focused on these two definitions of intelligence as our coding criteria, because, first, prior research finds that lay individuals in both cultures typically characterize intelligence in these terms^16,17^ and, second, prior research has indicated that individuals also show distinct malleability beliefs about fluid vs. crystallized intelligence^18^.

We chose effort/ability attribution as our academic motivational outcome. Prior research found a tight relation in which a more malleable view of intelligence was associated with attributing academic failure more to effort than ability^33^. In addition, people can have distinct effort/ability attributions for expert-level vs. school-level academic achievements (e.g., becoming a mathematics professor vs. getting an A in a middle-school level class^18^). Thus, Study 2 probed participants’ effort/ability attributions for both levels of academic achievement.

Study 2 addressed two main questions: (1) as a conceptual replication of Study 1, do Chinese and US students show qualitative differences in (a) intelligence mindsets and (b) how mindsets are associated with effort-ability attributions for achievements? Further, (2) how might variations in self-definitions of intelligence explain the cross-cultural differences in mindsets?

### Participants

Participants were college students recruited from the University of Michigan in the United States and Beijing Normal University in China. Both universities were considered one of the most highly selective public universities in the country. The US sample had *N* = 189, *M* (SD)_Age_ = 18.91 (1.28). The US participants were mostly first-year students (62.4%) and sophomores (26.5%). The Chinese sample had *N* = 171, *M* (SD)_Age_ = 19.09 (1.83). The Chinese participants were mostly first-year students (28.1%), sophomores (43.3%), and juniors (24.6%). Both samples included participants from a wide range of majors (including Science, Technology, Engineering, and Mathematics (STEM), social sciences, business, health sciences, etc.). The US sample was recruited from the university’s introductory psychology subject pool and completed the study for class credit, and the Chinese sample was recruited via online social media platforms and received $2.00 for their participation. The study was approved as exempt from further oversight by the University of Michigan Institutional Review Board (IRB # HUM00121520). All participants provided informed consent before entering the survey.

### Measures

#### Spontaneous intelligence definition

An open-ended question was administered asking participants to spontaneously define intelligence “based on your own understanding with your own words”. Each response was later coded as belonging to one of four categories: the statement reflects “only fluid intelligence”, “only crystallized intelligence”, “both fluid and crystallized intelligence”, or “neither fluid nor crystallized intelligence”. Two native speakers of each language respectively coded responses of each sample. For the US sample, the two coders had 82.2% agreement in their codes among all definitions, interrater reliability *κ* = 0.64; for the Chinese sample, the two coders had 87.6% agreement, *κ* = 0.74. These interrater reliability values suggest a substantial level of agreement^34^. Discrepancies were addressed as a group (all coders and the first author). A detailed coding protocol can be found in Supplementary Note 1.

#### Mindset of intelligence

Participants’ malleability beliefs about intelligence were assessed by the ITIQ^10^, an eight-item questionnaire with four reverse-coded items. Participants answer each item on a seven-point likert scale (1-Strongly disagree, 7-Strongly agree). One sample item is “You have a certain amount of intelligence, and you can’t really do much to change it”. The Chinese version was developed through a back-translation process.

#### Effort-ability attribution

Four questions were asked with regard to the relative importance of effort vs. ability on expert- and school-level mathematics achievements. The two expert-level achievements included “becoming a mathematics professor” and “winning the Fields award (equivalent to a Nobel prize in Mathematics)”, and the school-level ones were “achieving an A in a middle-school level algebra course” and “learning multiplication tables, fraction, and percentage operations”. For each question, participants answered from 1-“Pure ability” to 7-“Pure effort”, with 4 labeled as “Equally important”. The same measure was used in previous research^18^.

#### Mindset of fluid and crystallized intelligence

We used the same measure as in previous research^17^. Definitions of crystallized and fluid intelligence were presented to participants. After each definition, a modified ITIQ was presented with the term “intelligence” replaced by “fluid intelligence” or “crystallized intelligence”. According to the prior investigation^18^, these modified scales demonstrated high internal consistencies, validity, and were proven to measure separated constructs from the general ITIQ. These measures were presented after probing participants’ general intelligence mindset.

### Procedure

Participants entered a questionnaire via Qualtrics. They read a description of the study, completed the consent procedure, and proceeded to the questionnaire. The questionnaire had four blocks. Block 1 asked participants to define intelligence based on their own understanding and assessed their mindsets of intelligence. Block 2 first presented definitions of fluid and crystallized intelligence (randomized order), followed by the mindset items of each definition. Block 3 probed participants’ effort-ability attributions to different achievements. Finally, Block 4 collected participants’ demographic information.

### Reporting summary

Further information on research design is available in the Nature Research Reporting Summary linked to this article.

## Supplementary information

Supplementary Information

Reporting Summary

## Data Availability

Study 1 data can be downloaded from https://www.oecd.org/pisa/data/2018database/. Study 2 data are available to the public on Open Science Framework 10.17605/OSF.IO/37TUD.

## Code availability

Codes are available to the public on Open Science Framework 10.17605/OSF.IO/37TUD.
