# Supplementary material for: Growth mindset and academic outcomes: a comparison of US and Chinese students
Source: NPJ Sci Learn. 2021 Jul 19;6:21. doi: 10.1038/s41539-021-00100-z (PMC8290023; doi:10.1038/s41539-021-00100-z)
Supplement: Supplementary file 1 — Supplementary Information [file 41539_2021_100_MOESM1_ESM.pdf]

Supplementary Materials to manuscript

Growth mindset and academic outcomes: A comparison of US and Chinese students

Xin Sun*[1], Shaylene Nancekivell[2], Susan A. Gelman[1], & Priti Shah[1]

1 Department of Psychology, University of Michigan, 530 Church Street, Ann Arbor MI, 48109

2 Department of Psychology, University of North Carolina Greensboro, 1100 W Market St, Greensboro NC, 27403

Study 1 Figures *PISA mathematics scores by mindset responses by country*

PISA uses 10 plausible values to represent mathematics performance for each student (item labels appeared in the dataset: *PV1MATH* to *PV10MATH*) (OECD, 2009). In the manuscript, our Figure 1 reported score distribution by mindset for plausible value 1. Here, we attach the score distribution for all remaining plausible values.

Supplementary Figure 1:

Distribution of mathematics score (plausible values 2-10) by mindset response in the Chinese (B-J-S-G) sample

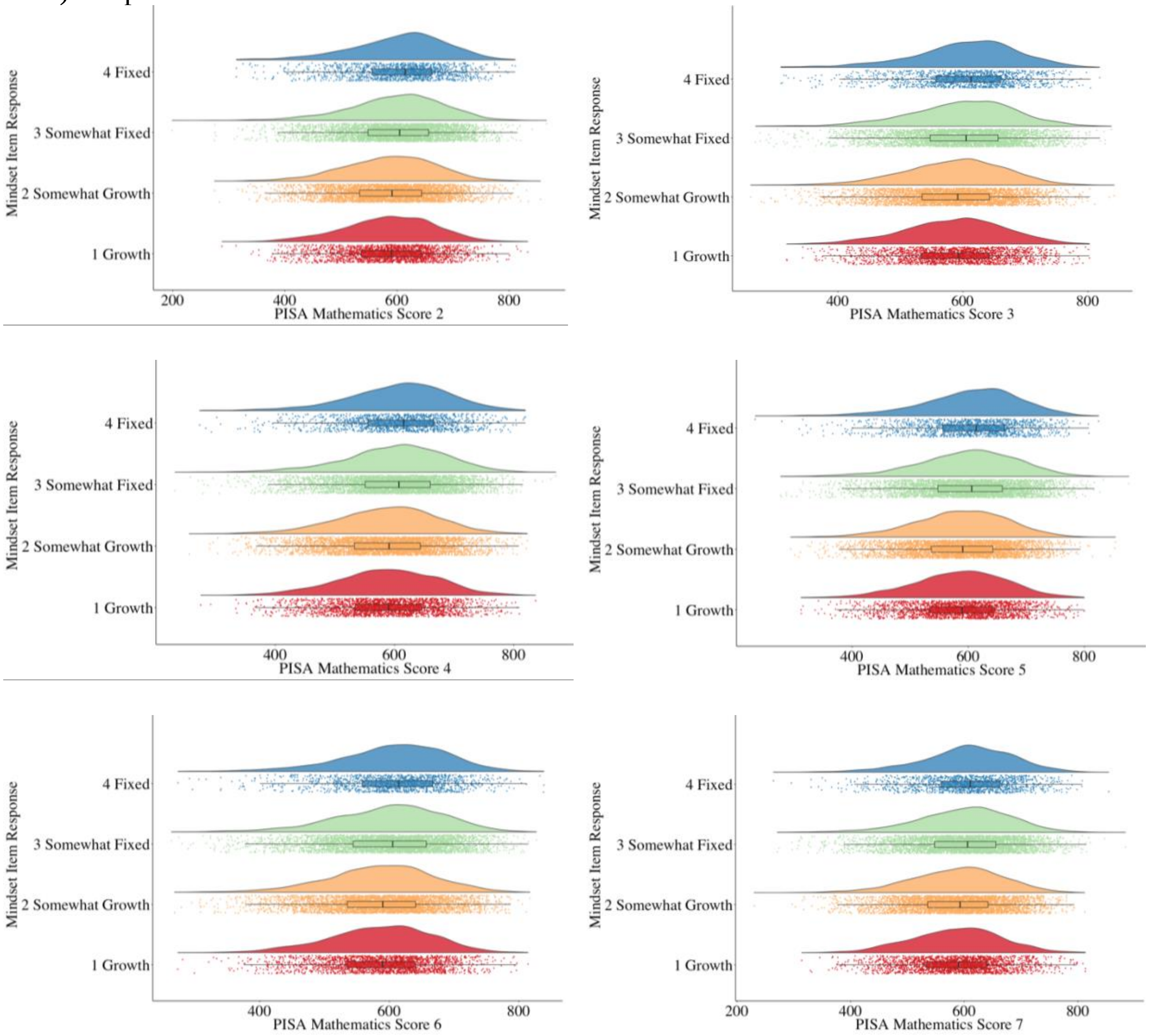

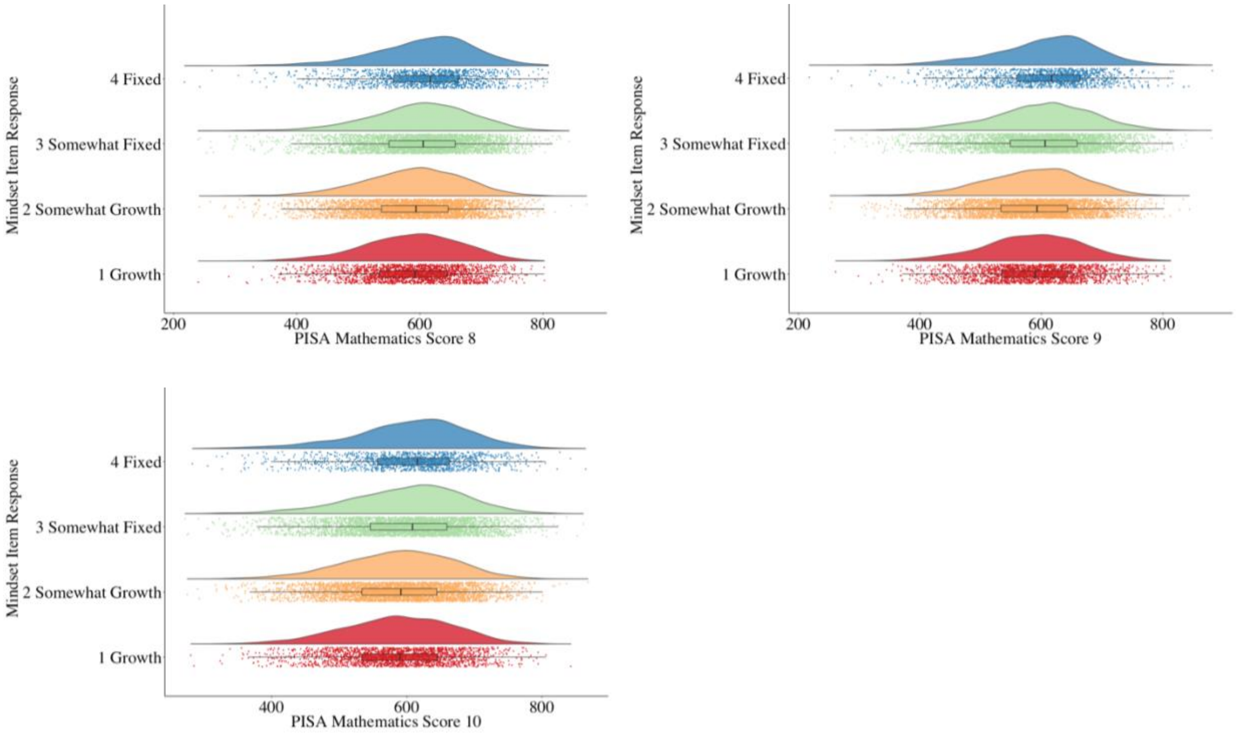

Supplementary Figure 2:
Distribution of mathematics score (plausible values 2-10) by mindset response in the US sample

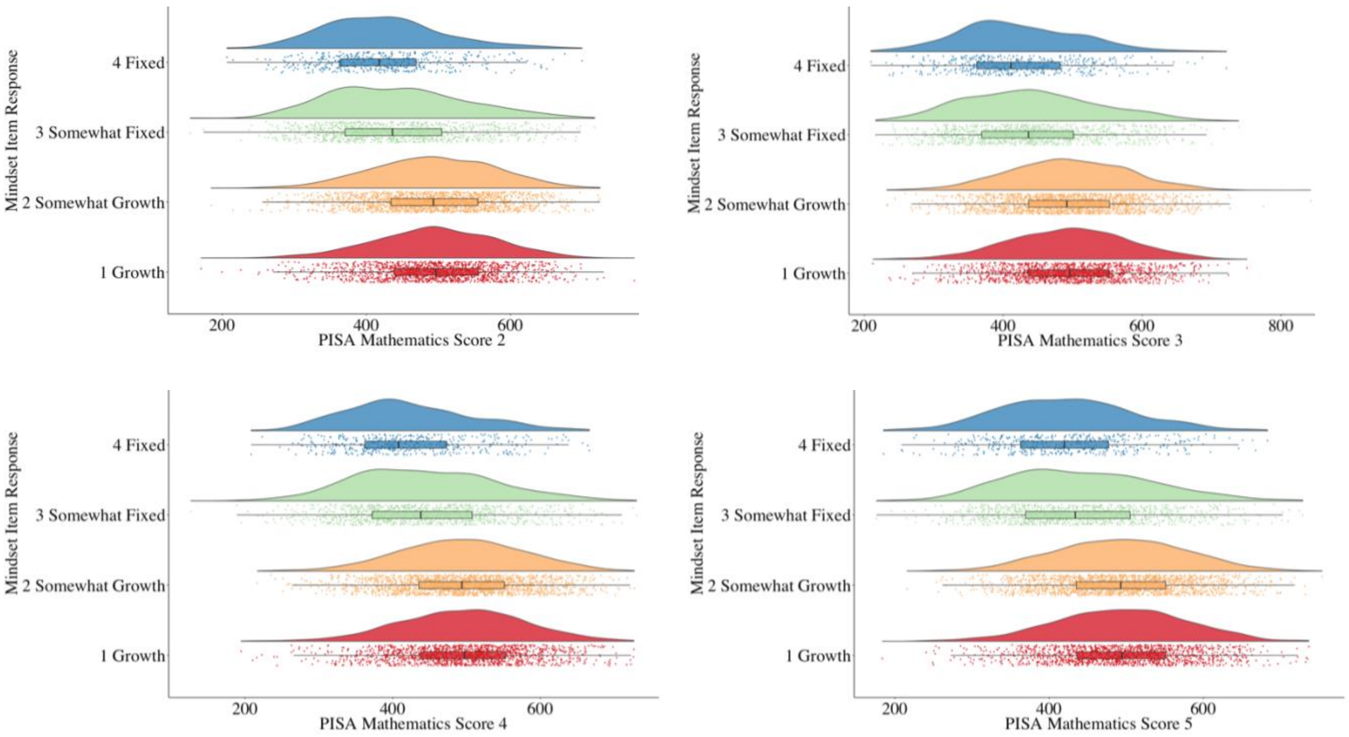

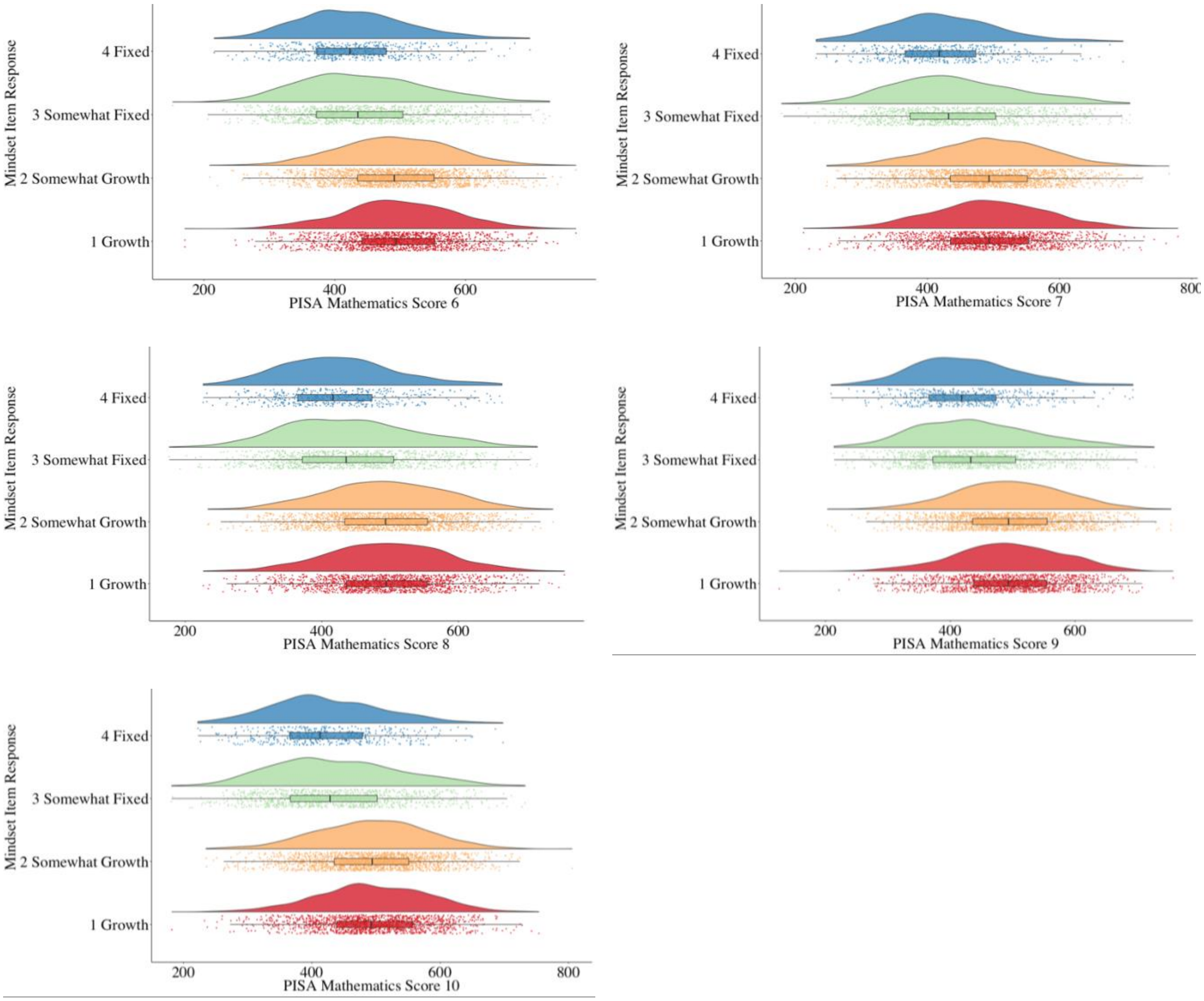
Mindset Item Response
4 Fixed
3 Somewhat Fixed
2 Somewhat Growth
1 Growth
200
400
600
800
PISA Mathematics Score 6
PISA Mathematics Score 7
PISA Mathematics Score 8
PISA Mathematics Score 9
PISA Mathematics Score 10

Supplement Note 1
Study 2 Coding protocol of intelligence definition

Coding Book: Intelligence

Introduction and Background

This coding scheme concerns how participants answered a question about "intelligence" during our study. In this study, the participants answered questions about their beliefs on intelligence.

Here is what they saw:
How would you describe **intelligence**? Please define **intelligence** based on your own understanding with your own words.

Coding Scheme

Your job will be to blind code the answers to this definition question. There will be two people coding all of the responses. Do not speak to the other coder during this process.

PART ONE: First, you will code whether or not the response was informative (1 = informative, 0 = uninformative). If the response is deemed uninformative do not code it further. Remember to only code what the participant actually says. For example, "Intelligence is a level of mental activity that can be measured through an IQ scale." is uninformative because we don't know what is the "IQ scale" in their mind.

Once you are done classifying the uninformative items let Xin know immediately. She will then resolve any disagreements between you and the other coder. Once those are resolved and she returns the spreadsheet to you, you can continue coding.

Example Uninformative Responses
"Intelligence is IQ."
"I don't know."

PART TWO: Based on the definitions and notes below, you will then code participants' responses as to whether it fits "Fluid Intelligence" and "Crystallized Intelligence". You should first code all statements for one category, then code all statements again for the other category. Note that the two categories are independent. This is to say, a statement can fit into both of them, or one but not the other, or neither of them.

1) **Fluid intelligence.**
   Fluid intelligence is defined as "how efficiently and accurately you solve problems, use logic, and identify patterns. For example, you would use fluid intelligence to identify which number goes next in a series (1, 2, 4, 7, 11, ...)"

   Note: fluid intelligence emphasizes **novelty in a problem (e.g., new or unfamiliar situations)** and it is **minimally based on prior knowledge/skills.**

Keywords/phrases clearly associated with fluid intelligence include:
adapt (to new/novel situations), understand (comprehend, process), explain, problem-solving, logic, analytical, abstract

2) **Crystallized intelligence**
Crystallized intelligence is defined as "your knowledge and skills such as vocabulary, common knowledge like knowledge of how to divide fractions, the names of the planets, or the location of different countries."
Note: crystallized intelligence is **experience-dependent**, it focuses on possessing learned knowledge/skills, and/or applying learned knowledge/skills to specific domains.

Keywords/phrases clearly associated with crystallized intelligence include:
have/apply/learn (knowledge, skills, facts, information, etc.), recall, acquire, memorize
Note: the keyword *learn* is usually deemed as crystallized, unless it is further framed in a fluid manner, for example, learn to connect ideas, learn to solve problems.

Important common notes:

- **Neutral keywords/phrases:**
  Some keywords don't really indicate fluid or crystallized intelligence on their own, they may include: ability/capacity and decision making.

  Why are these neutral?
  Decision-making -- we don't know if they mean "decide based on some prior knowledge," or "decide based on some kind of inductive or deductive reasoning process."
  Ability/capacity -- again, we are not sure if they mean to "learn, process information, reason", or "possess knowledge".

- **NEITHER keywords/phrases:**
  Some keywords may indicate neither CI nor FI. Neither type might include: music, interpersonal, emotional, creativity (like "thinking outside of the box"), or physical/athletic ability and something else. This category also includes statements with no clear meaning, for example, "smartness", because we don't know what participants mean when they say "smartness".

  These are all either not specifically cognitive (e.g., music, emotion), or creativity. These are all individual differences.

Supplementary References

OECD. *PISA 2018 Assessment and Analytical Framework* (OECD Publishing, Paris, 2019). https://doi.org/10.1787/b25efab8-en
