# Supplementary material for: Growth mindset and academic outcomes: a comparison of US and Chinese students
Source: NPJ Sci Learn. 2021 Jul 19;6:21. doi: 10.1038/s41539-021-00100-z (PMC8290023; doi:10.1038/s41539-021-00100-z)
Supplement: Supplementary file 2 — Reporting Summary [file 41539_2021_100_MOESM2_ESM.pdf]

nature research

Corresponding author(s): Xin Sun

Last updated by author(s): Jun 14, 2021

Reporting Summary

Nature Research wishes to improve the reproducibility of the work that we publish. This form provides structure for consistency and transparency in reporting. For further information on Nature Research policies, see our Editorial Policies and the Editorial Policy Checklist.

Statistics

For all statistical analyses, confirm that the following items are present in the figure legend, table legend, main text, or Methods section.

n/a	Confirmed	
☐	☒	The exact sample size (n) for each experimental group/condition, given as a discrete number and unit of measurement
☐	☒	A statement on whether measurements were taken from distinct samples or whether the same sample was measured repeatedly
☐	☒	The statistical test(s) used AND whether they are one- or two-sided<br>*Only common tests should be described solely by name; describe more complex techniques in the Methods section.*
☐	☒	A description of all covariates tested
☐	☒	A description of any assumptions or corrections, such as tests of normality and adjustment for multiple comparisons
☐	☒	A full description of the statistical parameters including central tendency (e.g. means) or other basic estimates (e.g. regression coefficient) AND variation (e.g. standard deviation) or associated estimates of uncertainty (e.g. confidence intervals)
☐	☒	For null hypothesis testing, the test statistic (e.g. F, t, r) with confidence intervals, effect sizes, degrees of freedom and P value noted<br>*Give P values as exact values whenever suitable.*
☒	☐	For Bayesian analysis, information on the choice of priors and Markov chain Monte Carlo settings
☐	☒	For hierarchical and complex designs, identification of the appropriate level for tests and full reporting of outcomes
☐	☒	Estimates of effect sizes (e.g. Cohen's d, Pearson's r), indicating how they were calculated

Our web collection on statistics for biologists contains articles on many of the points above.

Software and code

Policy information about availability of computer code

Data collection	N/A
Data analysis	N/A

For manuscripts utilizing custom algorithms or software that are central to the research but not yet described in published literature, software must be made available to editors and reviewers. We strongly encourage code deposition in a community repository (e.g. GitHub). See the Nature Research guidelines for submitting code & software for further information.

Data

Policy information about availability of data

All manuscripts must include a data availability statement. This statement should provide the following information, where applicable:

- Accession codes, unique identifiers, or web links for publicly available datasets
- A list of figures that have associated raw data
- A description of any restrictions on data availability

Study 1 data can be downloaded from https://www.oecd.org/pisa/data/2018database/. Study 2 data will be available to the public on Open Science Framework upon publication.

Field-specific reporting

Please select the one below that is the best fit for your research. If you are not sure, read the appropriate sections before making your selection.

- [ ] Life sciences
- [x] Behavioural & social sciences
- [ ] Ecological, evolutionary & environmental sciences

For a reference copy of the document with all sections, see nature.com/documents/nr-reporting-summary-flat.pdf

Behavioural & social sciences study design

All studies must disclose on these points even when the disclosure is negative.

Study description	Study 1 is a quantitative study. Study 2 mostly uses quantitative analyses, except for one piece of analysis that utilized a mixed method approach (i.e., coded participants statements into categories for subsequent quantitative analyses).
Research sample	Study 1 examined data from the US and Chinese samples of PISA 2018 (https://www.oecd.org/pisa/data/2018database/.). The US sample included N = 4,663 participants from 175 schools across the country (49.6% girls), excluding those with missing values (N = 175). For the Chinese sample, we included those students from mainland China, specifically from the provinces of Beijing, Jiangsu, Shanghai, and Guangdong (B-J-S-G). Taking out missing values (N = 79), the final Chinese sample included N = 11,979 participants from 362 schools (47.9% girls).<br>Study 2 participants were college students recruited from a Midwest university in the US and a university from Beijing, China. Both universities were considered as one of the most highly selective public universities in the country. The US sample had N = 189, with an age M(SD) = 18.91 (1.28). The US participants were mostly first-year students (62.4%) and sophomores (26.5%). The Chinese sample had N = 171, with an age M(SD) = 19.09 (1.83). The Chinese participants were mostly first-year students (28.1%), sophomores (43.3%), and juniors (24.6%). Both samples included participants from a wide range of majors (including STEM, social sciences, business, health sciences, etc.). The US sample was recruited from the introductory psychology subject pool and completed the study for class credit, and the Chinese sample was recruited online and received $2.00 for their participation.<br><br>Study 1 samples are very large in their sizes and demonstrate excellent representativeness. Study 2 samples were good representatives of college students in both countries.
Sampling strategy	Study 1: PISA sampling strategy can be found at: https://www.oecd.org/pisa/pisaproducts/SAMPLING-IN-PISA.pdf. PISA follows the stratified sampling approach.<br>Study 2: Study 2 used two convenient samples. Study 2 carefully chose the two universities to make sure their students' responses are comparable to each other (i.e., the two universities were both one of the highly selective public universities in each country). In terms of sample sizes, we referred to a paper with a preregistered study that used identical measures (Sun, Nancekivell, Gelman, & Shah, 2020).
Data collection	Study 1: Students participating in PISA complete the two-hour assessment on computer. Reference: https://www.oecd-ilibrary.org/education/pisa-2018-results-volume-i_5f07c754-en<br>Study 2: Students complete the questionnaire online anonymously using their own devices.
Timing	Study 1: year of 2018; Study 2: 2019 fall semester (Sept.-Dec.).
Data exclusions	Study 1: missing data was excluded (the US sample N = 175; the Chinese sample: N = 79). Study 2: data was excluded if participants failed to pass the attention check items.
Non-participation	Study 1: PISA 2018 school participation rates were 76.4% in the US and 99.4% in China (https://nces.ed.gov/surveys/pisa/2018technotes-11.asp#:~:text=In%20the%20United%20States%2C%20136,response%20rate%20of%2076%20percent.)<br>Study 2: No known dropouts or declines.
Randomization	There were no experimental manipulations in these studies.

Reporting for specific materials, systems and methods

We require information from authors about some types of materials, experimental systems and methods used in many studies. Here, indicate whether each material, system or method listed is relevant to your study. If you are not sure if a list item applies to your research, read the appropriate section before selecting a response.

Materials & experimental systems

n/a	Involved in the study	
☒	☐	Antibodies
☒	☐	Eukaryotic cell lines
☒	☐	Palaeontology and archaeology
☒	☐	Animals and other organisms
☐	☒	Human research participants
☒	☐	Clinical data
☒	☐	Dual use research of concern

Methods

n/a	Involved in the study	
☒	☐	ChIP-seq
☒	☐	Flow cytometry
☒	☐	MRI-based neuroimaging

Human research participants

Policy information about studies involving human research participants

Population characteristics	See above
Recruitment	For recruitment method, please see above sections.<br>Potential biases:<br>Study 1: according to the sampling manual from PISA, data from US and China are both with fair response rate and subject to relatively small biases (https://www.oecd.org/pisa/pisaproducts/SAMPLING-IN-PISA.pdf).<br>Study 2: We don't see any potential self-selection biases because the questionnaire was answered in an anonymous way and it did not involve strong incentives.
Ethics oversight	Study 2 was approved as exempt by the institutional review board in the first author's institution of affiliation.

Note that full information on the approval of the study protocol must also be provided in the manuscript.
